# Extrinsic Mortality Can Shape Life-History Traits, Including Senescence

**DOI:** 10.1007/s11692-018-9458-7

**Published:** 2018-06-13

**Authors:** Maciej J. Dańko, Oskar Burger, Krzysztof Argasiński, Jan Kozłowski

**Affiliations:** 1 Max Planck Institute for Demographic Research, Rostock, Germany; 2 Institute of Mathematics, Polish Academy of Sciences, Warsaw, Poland; 3 Institute of Environmental Sciences, Jagiellonian University, Krakow, Poland

**Keywords:** Selection gradients, Density-dependence, Resource allocation, Williams hypothesis, r/K selection, Fitness measures, Malthusian parameter, Net reproductive rate, Reproductive value

## Abstract

The Williams’ hypothesis is one of the most widely known ideas in life history evolution. It states that higher adult mortality should lead to faster and/or earlier senescence. Theoretically derived gradients, however, do not support this prediction. Increased awareness of this fact has caused a crisis of misinformation among theorists and empirical ecologists. We resolve this crisis by outlining key issues in the measurement of fitness, assumptions of density dependence, and their effect on extrinsic mortality. The classic gradients apply only to a narrow range of ecological contexts where density-dependence is either absent or present but with unrealistic stipulations. Re-deriving the classic gradients, using a more appropriate measure of fitness and incorporating density, shows that broad ecological contexts exist where Williams’ hypothesis is supported.

**Electronic supplementary material:**

The online version of this article (10.1007/s11692-018-9458-7) contains supplementary material, which is available to authorized users.

## Introduction

An enormous diversity of lifespans exists in nature, yet efforts to explain it remain inconclusive. The famous hypothesis by Williams (1957) that “low adult death rates should be associated with low rates of senescence, and high adult death rates with high rates of senescence”, is usually interpreted as a prediction that higher extrinsic mortality promotes either the earlier onset or a faster rate of senescence (e.g. Abrams 1993). Empirical tests of this hypothesis reveal both support and contradiction, as summarized by Furness and Reznick (2017; see also Ricklefs and Scheuerlein 2001; Williams et al. 2006; Ricklefs 2008; Pietrzak et al. 2015; da Silva 2018). This mixed support for Williams’ hypothesis has given rise to much theoretical debate, with some papers essentially rejecting it (Caswell 2007; Wensink et al. 2017), while others support the premise but limited to certain ecological scenarios (Abrams 1993; Cichoń 1997; Dańko et al. 2017; da Silva 2018). Theoretical papers by Abrams (1993) and Moorad and Promislow (2010) are often cited in this context to support the argument that the Williams’ hypothesis is essentially flawed, but this overlooks the fact that both papers show that adult death rates may or may not affect the rate of aging.

The choice of appropriate fitness measure is a key issue for any theoretical model. We show that assumptions inherent in the choice of fitness measure have had problematic effects for understanding the Williams’ hypothesis, and these problems relate to general discussions of how density and extrinsic mortality affect life history evolution. Most studies, including Hamilton’s selection gradients, use the Malthusian parameter, which is the solution of the Euler–Lotka equation (e.g. Caswell 2007; Caswell and Shyu 2017; Wensink et al. 2017). However, the Malthusian parameter is not the only measure of fitness and is not appropriate for all ecological situations. Specifically, the Malthusian parameter is well-suited for populations that are in phases of unconstrained growth or where density-dependence (DD hereafter) acts in such a way that it affects survival independently of age. However, DD may act on fertility or production rate, rather than just survival, or/and may have age-specific effects (Charnov 1990; Kozłowski 1993; Abrams 1993; Mylius and Diekmann 1995; Dańko et al. 2017). The choice of the Malthusian parameter as the fitness measure, and the narrow definition of DD it brings, are largely responsible for the confusion over Williams’ hypothesis. We modify selection gradients to reflect a more realistic ecological scenario that includes broader range of DD. We also identify the ecological contexts for when extrinsic age- or state-independent mortality affects senescence and other life history traits.

### Hamilton’s Indicators of the Force of Selection

Hamilton’s indicators of the force of selection (Hamilton 1966) are the formal foundation to the evolutionary theory of senescence (Abrams 1993; Rose et al. 2007; Ronce and Promislow 2010; da Silva 2018). These indicators show that selection pressure declines with age such that later ages are less important than earlier ones (Haldane 1941; Medawar 1946, 1952; Williams 1957). The three main theories of aging are based on this simple observation. These include: (i) mutation accumulation, where senescence results from late-acting deleterious germline mutations (Medawar 1952; but see Dańko et al. 2012; Dańko and Kozłowski 2012); (ii) antagonistic pleiotropy theory, where senescence results from a balance between benefits of mutations at early ages to the costs at later ages (Williams 1957; but see Maklakov et al. 2015); and (iii) disposable soma theory, where senescence results from tradeoffs between the allocation of resources to reproduction and somatic maintenance/repairs (Kirkwood 1977, formalized e.g. by Cichoń 1997; Drenos and Kirkwood 2005). Hamilton’s indicators of senescence also play a crucial role in quantitative genetics models (Lande 1982; Charlesworth 1990, 1994, 2001; Moorad 2014).

The fitness measure used by Hamilton to calculate his indicators is the Malthusian parameter (*r*), which is a solution of the Euler–Lotka equation:

1 $$1=\mathop \sum \limits_{{x=0}}^{\infty } {e^{ - rx}}{l_x}{m_x}$$

where *m*_*x*_ is the birth rate per capita for mothers of age *x* and *l*_*x*_ is the probability of surviving to age *x*. Hamilton considered mutations with effects on a given age class *a* and investigated how selection acts against them. The effect of mutations on survival or fertility was evaluated with the sensitivity of *r* to the natural logarithm of survival *p*_*a*_ (or negative hazard *µ*_*a*_):

2 $$\frac{{dr}}{{d{\text{ln}}{p_a}}}= - \frac{{dr}}{{d{\mu _a}}}=\frac{1}{T}\mathop \sum \limits_{{x=a+1}}^{\infty } {e^{ - rx}}{l_x}{m_x}$$

while the sensitivity of population growth rate to a mutation affecting fertility at age *a* as:

3 $$\frac{{dr}}{{d{m_a}}}=\frac{{{e^{ - ra}}{l_a}}}{T}$$

In both equations the age interval is assumed to be equal to 1 and *T* is defined as:

4 $$T=\mathop \sum \limits_{{x=0}}^{\infty } x{e^{ - rx}}{l_x}{m_x}$$

**Fig. 1:**
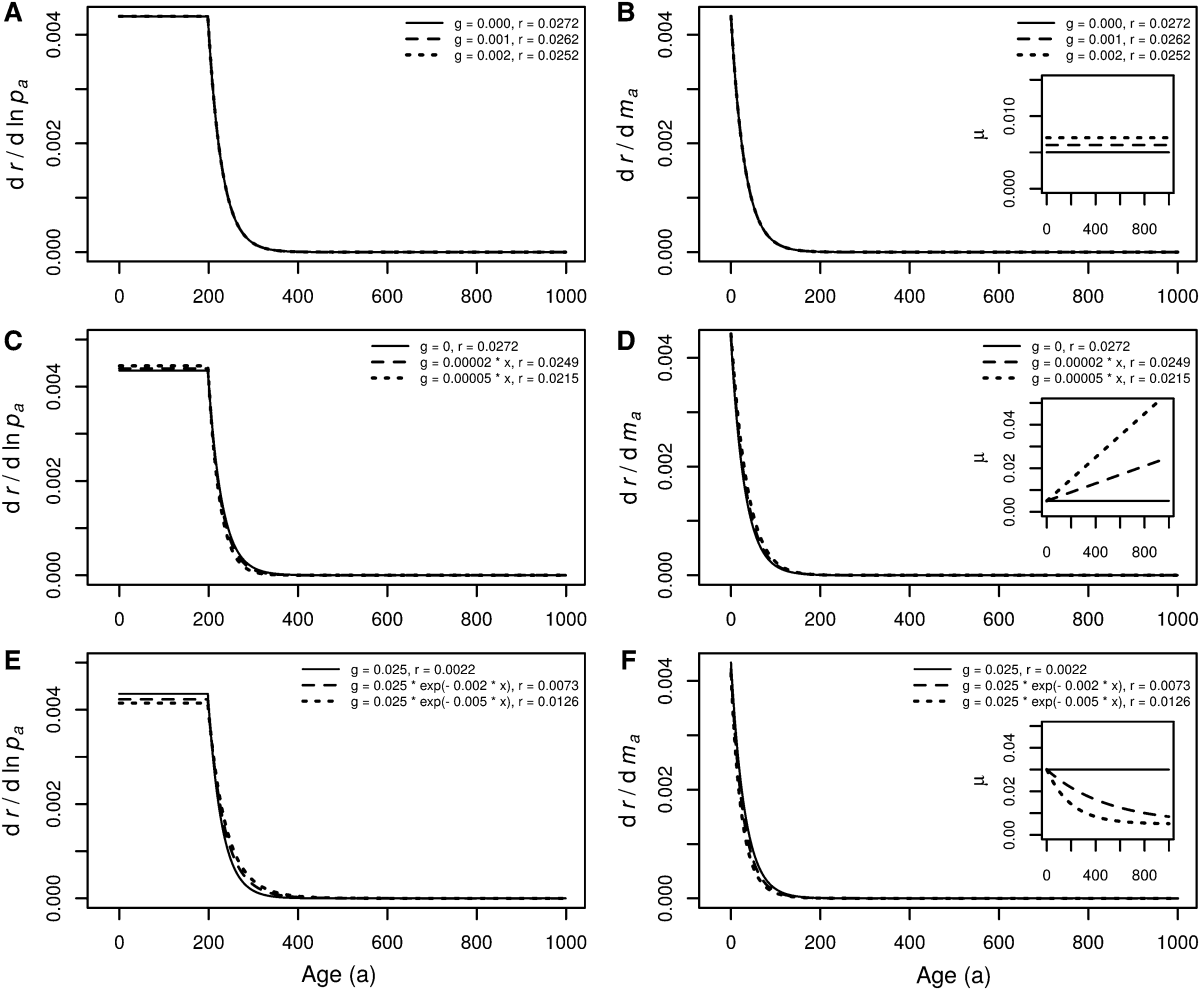
Selection gradients for *r* under different extrinsic mortalities. **a, b** age-independent extrinsic mortality, **c, d** extrinsic mortality increasing with age, and **e, f** extrinsic mortality decreasing with age. Before adding extrinsic mortality, each of the cases has the life-history defined in the same way: the background probability of surviving an age class *x* (e.g. measured in days) is constant and equal *p*_*x*_ = 0.995; the fertility *m*_*x*_ is 0 before maturity and 20 after maturity; maturity occurs at age 200. Insets show the total age-specific mortality calculated as $~{\mu _x}= - \ln {p_x}+g\left( x \right),$ where *g* is extrinsic mortality. The Malthusian parameter (*r*) is calculated from the Euler–Lotka equation, taking into account both background and extrinsic mortality

Both of these selection gradients decline monotonically with age even if there is no mortality (Hamilton 1966), as shown in Fig. 1a, b. This could imply that declining survivorship is not a prerequisite for declining gradients.

### In the World of Hamilton’s Indicators, William’s Hypothesis Does Not Work or Works Poorly

Declining selection pressure with age is related to the influential hypothesis about the effect of adult mortality on senescence proposed by Williams (1957). Although Williams’ hypothesis is widely quoted as a prediction, it was shown long ago that Hamilton’s declining selection gradients in fact make no clear theoretical prediction about the effect of changing mortality on ageing or lifespan. Typically, extrinsic mortality is considered to act uniformly with age, as follows, with age-independent hazard *τ* added to Eq. 1:

5 $$1=\mathop \sum \limits_{{x=0}}^{\infty } {e^{ - rx}}{e^{ - \tau x}}{l_x}{m_x}=\mathop \sum \limits_{{x=0}}^{\infty } {e^{ - \left( {r+\tau } \right)x}}{l_x}{m_x}~$$

Here, any increase of mortality by *τ* also decreases *r* exactly by *τ*, and in result *r* + *τ* is constant, meaning that changes in age-independent mortality have no effect on the selection gradients and hence any life history traits (Taylor et al. 1974; Abrams 1993; Charlesworth 1994; Caswell 2007; Moorad and Promislow 2008; Wensink et al. 2017; Dańko et al. 2017). Figure 1a, b illustrates such a situation and Fig. 1c–f shows that the gradients change only slightly even if extrinsic mortality varies with age. Thus, simply changing to more complicated mortality schedules does not seem to be enough to fix the predictive problem about the role of mortality stemming from Hamilton’s selection gradients. Further, *dr*/*dp*_*a*_ and *dr*/*dm*_*a*_, change in the opposite direction if mortality is age-dependent, which may partly neutralize the effect of age-dependent mortality. Qualitatively similar results were obtained under the more realistic assumption that mutations affect not only age *a*, but also all subsequent ages (see Supplementary Materials).

Hamilton’s force of selection declines rapidly with age. According to Medawar’s hypothesis, deleterious mutations expressing late in life should accumulate as a result. Similar logic applies to antagonistic pleiotropy (Rose et al. 2007). The rate of accumulation of these late-age deleterious mutations is not related to age-independent mortality and is only weakly related to age-dependent mortality. Because Hamilton’s force of selection relies on the Malthusian parameter *r*, the conclusions can only be drawn for the world described by this very specific fitness measure. However, as we show below, r is not the appropriate fitness measure for the most relevant ecological contexts.

### Selection Gradients Must Be Calculated for the Appropriate Fitness Measure

Hamilton’s indictors of selection are derived using the fitness measure *r*. However, the Malthusian parameter is not an appropriate measure of fitness for most forms of DD (Kozłowski 1993, 1999; Mylius and Diekmann 1995; Roff 2008). Evolution by natural selection requires competition among phenotypes. For this to occur the underlying traits must exhibit variation. As a result, fitness measures should allow for the examination of competing (variable) traits and strategies. Evolutionarily Stable Strategy (ESS) approaches are considered the master fitness criterion precisely because of this need to study competing strategies (Maynard Smith and Price 1973; Metz et al. 1992, 2008; Roff 2008; Dańko et al. 2017). While finding solutions to ESS models may require somewhat complex numerical methods, they can sometimes be simplified to basic fitness functions. Specifically, if DD acts on survival uniformly with age, the appropriate measure of fitness is the Malthusian parameter (*r*) measured at negligible densities. Alternatively, if DD acts through juvenile mortality/migration or fecundity (uniformly with age) and the population has a stable age structure, expected lifetime offspring production measured at negligible densities (*R*_0_), defined as:

6 $${R_0}=\mathop \sum \limits_{{x=0}}^{\infty } {l_x}{m_x},$$

is the appropriate measure of fitness (e.g. Kozłowski 1993; Mylius and Diekmann 1995).

Current studies on competing strategies involve replicator dynamics (RD) operating simultaneously on frequencies of multiple strategies. The role of DD is examined by combining the dynamics of changing population size with the dynamics of changing frequencies in the mixture of strategies (Argasinski and Broom 2017a, b). The RD confirm that the winning life history strategies are those that maximize expected lifetime offspring production (*R*_0_) when DD either (i) acts on the recruitment probability of juveniles or, (ii) suppresses birth rate to make the population stationary (Argasinski and Broom 2013; Argasinski and Rudnicki 2017; Rudnicki 2017). When the steady state contains a mixture of *R*_0_ maximizing strategies that have different expected lifespans (different mortalities), then any decrease of the population size or invasion of other suboptimal strategies will induce selection among *R*_0_ maximizers toward strategies with shorter expected lifespan (and shorter generation time) (Argasinski and Broom 2013; Argasinski and Rudnicki 2017). These results give further support to the claim that *R*_0_ is a more appropriate measure of fitness than the Malthusian *r* for a broad range of ecological scenarios.

### Selection Gradients Are Different When Fitness is Measured by Lifetime Offspring Production (*R*_0_)

Hamilton’s indicators should be based on *R*_*0*_ if the research question pertains to density-dependent populations regulated by emigration/death of juveniles or by age-independent effects on fertility. The sensitivity of *R*_0_ to any mutation affecting the natural logarithm of survival *p*_*a*_ at age class *a* is the remaining net reproductive success calculated from the next age class:

7 $$\frac{{d{R_0}}}{{d{\text{ln}}{p_a}}}= - \frac{{d{R_0}}}{{d{\mu _a}}}=\mathop \sum \limits_{{x=a+1}}^{\infty } {l_x}{m_x}$$

The sensitivity of *R*_0_ to a mutation affecting fertility is survivorship at age *a*:

8 $$\frac{{d{R_0}}}{{d{m_a}}}={l_a}$$

**Fig. 2:**
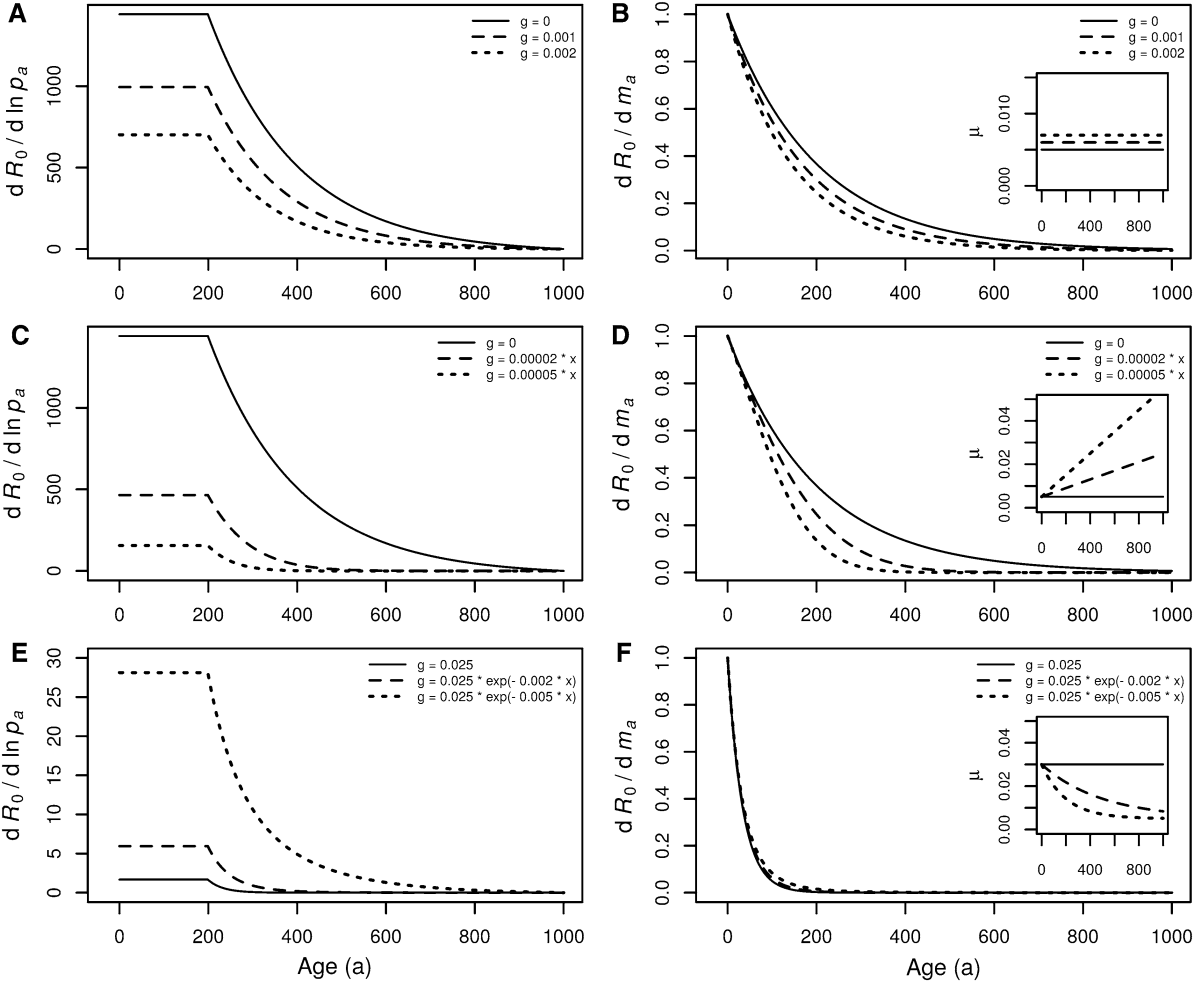
Selection gradients for *R*_*0*_ under different extrinsic mortalities. **a, b** age-independent extrinsic mortality, **c, d** extrinsic mortality increasing with age, and **e, f** extrinsic mortality decreasing with age. Each panel is characterized by the same *l*_*x*_ and *m*_*x*_ vectors as corresponding panel in Fig. 1. Insets show the total age-specific mortality calculated as $~{\mu _x}= - {\text{ln}}{p_x}+g(x)$, where *g* (extrinsic mortality) is delivered in figures’ legends. For further details see description of Fig. 1

The shape of these selection gradients based on *R*_0_ are similar to the corresponding Hamilton gradients. However, the additional age-independent extrinsic mortality is not compensated by a decrease in *r* because it is simply not present in the equations. This means that when *R*_0_ is maximized, extrinsic mortality plays a significant role in shaping life histories (Fig. 2a, b) (see also Kozłowski 1992, 1999, 2006; Charnov 1991, 1993; Cichoń 1997; Kozłowski et al. 2004; Dańko et al. 2012, 2017; Dańko and Kozłowski 2012; da Silva 2018). Furthermore, when extrinsic mortality increases (Fig. 2c, d) or decreases (Fig. 2e, f) with age, both selection gradients change in the same direction (the gradients based on the Malthusian parameter change in opposite directions). In this scenario the Williams hypothesis is supported, which translates to the conclusion that extrinsic mortality, age-dependent or not, has a strong impact on aging.

Corresponding panels in Figs. 1 and 2 show different selection gradients for the same life histories (the same *l*_*x*_ and *m*_*x*_). Gradients based on the Malthusian parameter (Fig. 1) decline much more steeply than the gradients based on *R*_0_. As such, in the world where *r* is fitness, senescence should start very soon after maturation. In the world when *R*_0_ is fitness, the onset of senescence may be delayed or advanced depending on external mortality.

### It’s Time to Revisit *r*- and *K*-Selection

The *r* and *R*_0_ fitness measures are tightly connected with the concepts of *r*- (world where *r* is a fitness measure) and *K*- (world where *R*_0_ is a fitness measure) strategies, which have a long history in ecology (MacArthur and Wilson 1967). Unfortunately, the usefulness of these concepts was corrupted by the association with specific sets of life history traits. This corruption was started unintentionally by Pianka (1970), who correctly enumerated traits of *r*-selected species, such as rapid development, early reproduction, and small size, as traits that lead to high *r* in Euler–Lotka equation (see also Kozłowski 2006; Dańko et al. 2018). Unfortunately, he assigned the opposite traits to *K*-selected species. In fact, these *r*-selected traits can also evolve in stable populations if mortality is high and DD acts on juvenile mortality or adult fecundity (Kozłowski 2006; Dańko et al. 2017). Originally, the classification to *r*-selected species meant that a population is almost always in an exponential growth phase, whereas classification to *K*-selected species meant that it is almost always in a steady-state under density-dependent regulation. Because the size of a population cannot increase exponentially for long, periods of population growth for *r*-selected species must be interspersed with major population crashes. After a crash, the few surviving individuals start the next period of rapid growth. In *r*-selected populations, the Malthusian parameter is a reasonable measure of fitness, and selection favors short generation time, which is accompanied by fast individual growth and small adult body mass. Hamilton’s indicators were constructed, probably unintentionally, for such populations as the Euler–Lotka equation was the most well-known and tractable definition of fitness (see also Lande 1982).

Because of the strong selection for short generation time in *r*-selected species, specific patterns of age-specific mortality or fecundity don’t have much of an effect on the life history, as illustrated in Fig. 1. Therefore, it is difficult to imagine *r*-selected species that are either long-lived or large. Fortunately, there is also a place in the world for *K*-selected species that can be small or large, short-lived or long-lived, depending on mortality. Because of these differences in how density dependence affects selection, it can be appropriate to maximize either *r* or *R*_0_ and this choice should be defined, and a rationale provided to support it.

There is another reason why the concepts of *r*- and *K*-selection, so popular in 1960s and 1970s, have fallen out of favor. These concepts were based on the classic logistic equation

9 $$\frac{{dn}}{{dt}}=r\left( {1 - \frac{n}{K}} \right)n$$

in which the ratio of density *n* over carrying capacity *K* affects *r* directly. Because *r* is defined as the difference between birth rate *b* and death rate *m* (*r*(*n*) = *b*(*n*) − *m*(*n*)), population density may affect *b, m* or both. Not specifying how DD acts on *b* and/or *m* precludes the possibility of using models based on Eq. (9) in studies of the effects of DD on life history traits (Kozłowski 1980; Argasinski and Kozłowski 2008). As shown in the next section, assumptions about DD must be much more carefully defined for studying the effect of extrinsic mortality on life history traits. The effect of density must be considered separately for birth rate and death rate, not for the difference between these two to make predictions on the evolution of life history traits.

### There is a Continuum of ESS Between the Maximization of *R*_0_ and *r* (or Between the Worlds of K- and r-Selection)

**Fig. 3:**
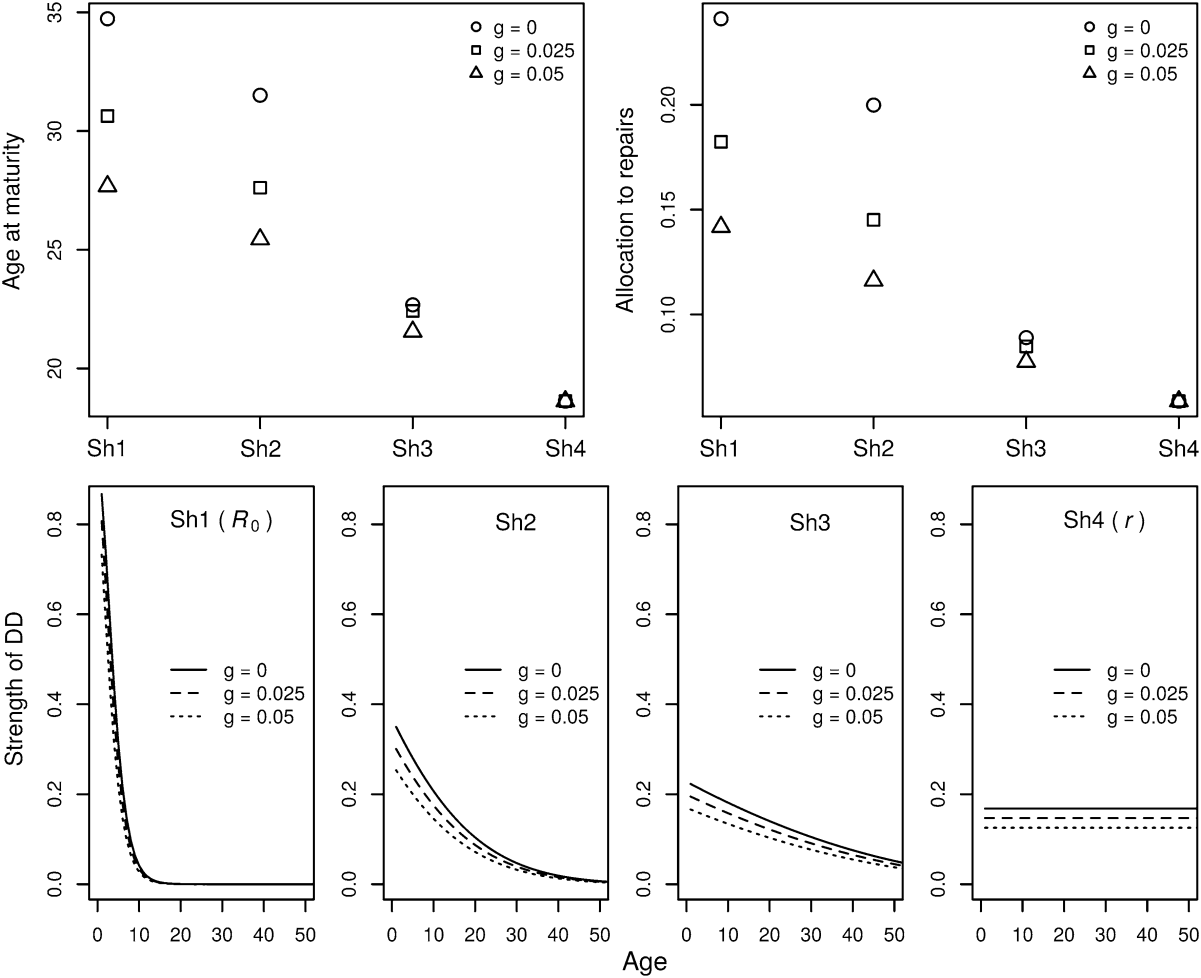
Evolutionarily stable life history strategies (upper panels) under different shapes of density dependence acting on survival (bottom panels). The results are extracted from Fig. 5 from (Dańko et al. 2017) ESS allocation model. It is assumed that an organism first allocates resource to growth and then switches completely to reproduction, while allocation to repairs is independent of age. The parameter *g* (*c* in Dańko et al. 2017 model) is age-independent extrinsic mortality as in Figs. 1 and 2. The Sh1–Sh4 represent different shapes of strength of DD, as illustrated in the bottom panels. In Sh1 DD acts only on juveniles (ESS can be obtained by maximization of *R*_0_), in Sh4 DD acts uniformly on all age classes (ESS can be obtained by maximization of *r*). Sh2 and Sh3 represent intermediate cases (maximization of neither *R*_0_ nor *r* gives ESS)

Dańko et al. (2017) showed that there is a continuum of different ESS strategies when DD acts on survival. The continuum has two boundaries obtained by maximization of *R*_0_ and *r* (both measured at negligible densities). The first boundary (*R*_0_ in Fig. 3) occurs when DD acts purely on survival of juveniles and does not affect survival of adults. The second boundary (*r* in Fig. 3) occurs when DD acts uniformly on all age classes (mathematically equivalent to the case when there is no density dependence). When the shape of DD is flat such that both juveniles and adults are affected, then (i) the effect of extrinsic mortality on life history becomes less apparent, (ii) and the age at maturity, size at maturity (not shown), and allocations to repair (determining onset and rate of senescence) decrease. The latter observation is anticipated by the observation made above that selection gradients decline faster for *r* than for *R*_0_ (Fig. 1 vs. Fig. 2). Thus, the study of extrinsic mortality’s influence on aging should not be separated from its effects on other life history traits, age at maturity in particular. Optimal resource allocation models, integrating demographic and physiological approaches, are promising tools for this purpose.

Dańko et al. (2017) showed that extrinsic mortality interacts with DD via a compensation mechanism, where high extrinsic mortality reduces population density and thus the strength of DD (Fig. 3, bottom row). Such compensation may be responsible for a great deal of the life history variation observed in the field or lab, because the offset is never complete except when DD acts uniformly on mortality rate or when DD does not work at all (such ideal cases are likely rare in nature). In addition to different age- or state-dependent patterns, DD may also act on different aspects of life: survival, fertility, or production. As a result, different life-histories may evolve even under the same extrinsic mortality. Furthermore, different types of DD cause extrinsic mortality to have different effects on age/size at maturity, growth rate, and allocations to repairs (Dańko et al. 2017).

### *r* in Fisher’s Reproductive Value is Not the Same as *r* in the Euler–Lotka Equation

Using *r* as a fitness measure is further complicated by the fact that there are two different “little *r*” that are sometimes wrongly used interchangeably. These “two *r*” include *r* from the Euler–Lotka equation (Eq. 1) and *r* from reproductive value *V*_*x*_:

10 $${V_x}=\frac{1}{{{l_x}}}\mathop \sum \limits_{{y=x}}^{\infty } {e^{ - r\left( {y - x} \right)}}{l_y}{m_y}$$

which reduces to reproductive value at birth when *x* = 0 :

11 $${V_0}=\mathop \sum \limits_{{x=0}}^{\infty } {e^{ - rx}}{l_x}{m_x}$$

In each of these equations *r* has a different meaning, yet they are often used interchangeably (e.g. Lande 1982; Caswell 2010; Wensink et al. 2017). This distinction is especially important when defining fitness in models for populations that contain a mixture of strategies. This issue was identified and resolved independently by Kawecki and Stearns (1993) and Houston and McNamara (1992), and summarized by Kozłowski (1993). A likely cause of the misunderstanding is the simple fact that *r* occurs in exp(− *rx*) in both the Euler–Lotka equation and *V*. However, in *V* the term exp(− *rx*) is a factor that discounts the value of future offspring by population growth. This discounting factor depends on population growth rate and is equivalent to the Malthusian *r* only when a single strategy exists. However, as mentioned above, evolution by natural selection requires variability, and if there are multiple strategies in the population these two *r* are no longer equivalent. When *r* in *V* equals the Malthusian parameter, then variation effectively does not exist. In the context of studying ESSs, *r* and the population discounting factor are initially equal to the Malthusian parameter of the resident strategy, but not the invader (e.g. Metz et al. 1992, 2008; Roff 2008; Dańko et al. 2017). If the population of the resident strategy is at equilibrium because of DD, the discounting factor equals 1, and *V*_0_ (reproductive value at birth) of the invader becomes equal to its lifetime offspring production *R*_0_ independently of its Malthusian parameter. Distinguishing between these two *r* is extremely important, because *V*_*0*_ is a universal fitness measure when different strategies compete (Taylor et al. 1974). We advocate using the symbol *r*_*p*_ (*p* for population) in reproductive value at birth *V*_0_ to avoid ambiguity.

## Concluding Remarks

Hamilton’s indicators of the force of selection are frequently used in evolutionary biology. Because they are based on the maximization of *r*, they only apply to ecological contexts characterized by unconstrained population growth or DD that acts uniformly on survival. The first case is likely to occur after a dramatic population collapse, when a small number of individuals find a new environment or survive for the next cycle. If such collapses are regular, traits adapting to the maximization of *r* can evolve (*r*-selection, short fast growth, small size, short life). Because most of the world experiences DD, such conditions are more likely to be found in a drop of water than in a tropical forest. That is, DD is likely common in nature and therefore its effects should not be ignored. If a small number of *K*-strategists survive population collapse or colonize a new place, selection maximizing lifetime offspring production *R*_0_ is temporarily suspended, but population growth cannot last long enough to redirect life history traits toward those of *r*-selected species. As DD operates, the direction of selection will be determined by the age-pattern and phenotypic effect of the DD processes. If DD acts on juvenile survival or fertility, then expected lifetime offspring production is maximized and extrinsic mortality will play a role in shaping the evolution of life history traits, including senescence, contrary to the world when *r* is maximized.

We hope the community of ecological theorists realizes that each of these fitness measures, *r* and *R*_0_, describe different worlds, but with a continuum between them. Extrinsic mortality plays a variable role on the evolution of life history traits, including senescence, and can be neglected only close to *r*-edge of this continuum. Researchers are correct in arguing that the Williams’ hypothesis about the effect of age-independent mortality on senescence should not be taken as a general ecological prediction (Caswell 2007; Moorad and Promislow 2010; Caswell and Shyu 2017; Wensink et al. 2017). However, we show that the converse statement, that extrinsic mortality has no effect on life history evolution, is definitely not universally true. Depending on the effects of DD, extrinsic mortality may or may not drive the timing or rate of senescence. Caswell and Shyu (2017, p. 65) showed that the selection gradient is unchanged by stage-independent mortality for any kind of age- or stage-classified demography and any form of stage-independent DD. While we agree, this statement about an unchanging selection gradient may have the unfortunate byproduct of amplifying the (incorrect) view that age-independent extrinsic mortality plays no predictive role in life history evolution. In fact, stage-independent DD, which works uniformly on mortality across all age classes, is an unlikely form of DD. DD that acts on fertility is a form of stage-dependent DD, because it only affects adults. Similarly, DD that acts uniformly on production my affect fertility in ways that vary with age. And of course, DD that acts on juveniles is, by definition, stage-dependent. Clearly, there is plenty of room for the effects of stage-independent extrinsic mortality on life histories in nature.

Mortality often depends on the age of individuals, as they become less able to move quickly and avoid predators, for example, and may depend on other condition-dependent attributes such as reproductive status, infection, or poor nutrition (Chen and Maklakov 2012; Dowling 2012). Such condition-dependent mortality is not random (with respect to age or stage) and can explain many of the observed exceptions to Williams’ hypothesis (Williams and Day 2003; Chen and Maklakov 2012; Dowling 2012; Furness and Reznick 2017; da Silva 2018). We strongly support further work on the role of condition-dependent mortality on the evolution of aging patterns and life history strategies. However, we add the caveat that condition-dependence should not be invoked unnecessarily, especially without considering the effects of stage-independent mortality.

We appeal to theoreticians to precisely describe the scope of applicability of their models, especially from the point of view of DD. For empirically oriented ecologists, it is extremely important to study DD in natural populations, as the precise form of DD is rarely known (Ginzburg et al. 2010). Such research was common in the 1960s, but mostly abandoned later. In comparative studies, when little is known about DD, predictions of models based on both *r* and *R*_0_ maximization should be compared. Indeed, the exciting part is that a great deal of effort remains for those interested in explaining the diversity of life histories. Theory based on *r*-maximization could be applied mainly to plants and animals that can be watched under microscopes, but not by binoculars.

## Electronic supplementary material

Below is the link to the electronic supplementary material.

Supplementary material 1 (DOCX 519 KB)
