# Supplementary material for: Extrinsic Mortality Can Shape Life-History Traits, Including Senescence
Source: Evol Biol. 2018 Jun 13;45(4):395–404. doi: 10.1007/s11692-018-9458-7 (PMC6223763; doi:10.1007/s11692-018-9458-7)
Supplement: Supplementary file 1 — Supplementary material 1 (DOCX 519 KB) [file 11692_2018_9458_MOESM1_ESM.docx]

# **Extrinsic mortality can shape life-histories, including senescence**

Maciej J. Dańko^1^,*, Oskar Burger^1^, Krzysztof Argasiński^2^ and Jan Kozłowski^3^

^1^ Max Planck Institute for Demographic Research. Rostock, Germany

^2^ Institute of Mathematics, Polish Academy of Sciences, Warsaw, Poland

^3^ Jagiellonian University, Institute of Environmental Sciences, Krakow, Poland

* Corresponding author

**Supplementary materials**

1. **Gradients of** *r* **for mutations affecting multiple ages**

The gradient of *r* for a mutation acting on ln *p_x_* form age *a* onwards is given by:

$\frac{dr}{d\ln p_{a,...,\infty}}=-\frac{dr}{d\mu_{a,...,\infty}}=\frac{1}{T}\sum_{x=a+1}^{\infty} (x-a)e^{-rx}l_{x}m_{x}$ or

$\frac{dr}{d\ln p_{a,...,\infty}}=\frac{1}{T}\int_{a}^{\infty} (x-a)e^{-rx}l_{x}m_{x}dx$ in the continuous form (Hamilton 1966),

The similar gradient of *r* for *m_x_* can be written as:

or

in the continuous form.


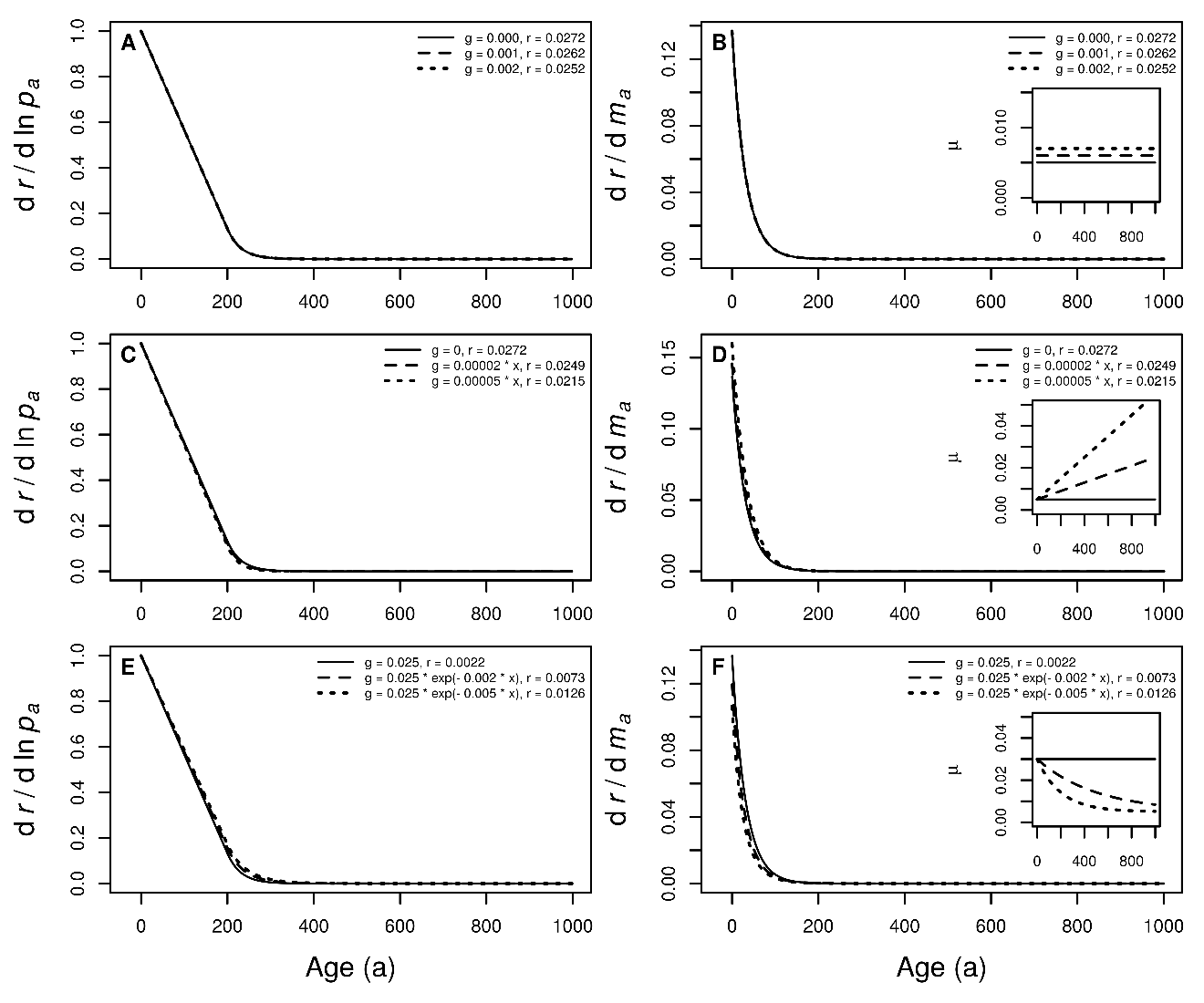


**Figure A1. Selection gradients for *r* under different extrinsic mortalities. A mutation affects age *a* onwards.** **AB** – age-independent extrinsic mortality, **CD** – extrinsic mortality increasing with age, and **EF** – extrinsic mortality decreasing with age. Before adding extrinsic mortality, each of the cases has the life-history defined in the same way: the background probability of surviving an age class *x* (e.g. measured in days) is constant and equal *p_x_* = 0.995; the fertility *m_x_* is 0 before maturity and 20 after maturity; maturity occurs at age 200. Inserts show the total age-specific mortality calculated as $\mu_{x}=-\text{ln}p_{x}+g\left( x \right),$ where *g* is extrinsic mortality. The Malthusian parameter (*r*) is calculated from the Euler-Lotka equation, taking into account both background and extrinsic mortality.

1. **Gradients of** *R_0_* **for mutations affecting multiple ages**

When *R_0_* is the measure of fitness the gradients of *R_0_* are calculated equivalently to gradients of *r.* For *p_x_* they are given as:

$\frac{dR_{0}}{d\ln p_{a,...,\infty}}=-\frac{dR_{0}}{d\mu_{a,...,\infty}}=\sum_{x=a+1}^{\infty} (x-a)l_{x}m_{x}$or

$\frac{dR_{0}}{d\ln p_{a,...,\infty}}=\int_{a}^{\infty} (x-a)l_{x}m_{x}dx$ in the continuous form.

And for *m_x_* as:

or

in the continuous form, where *e_a_* is life expectancy at age *a*


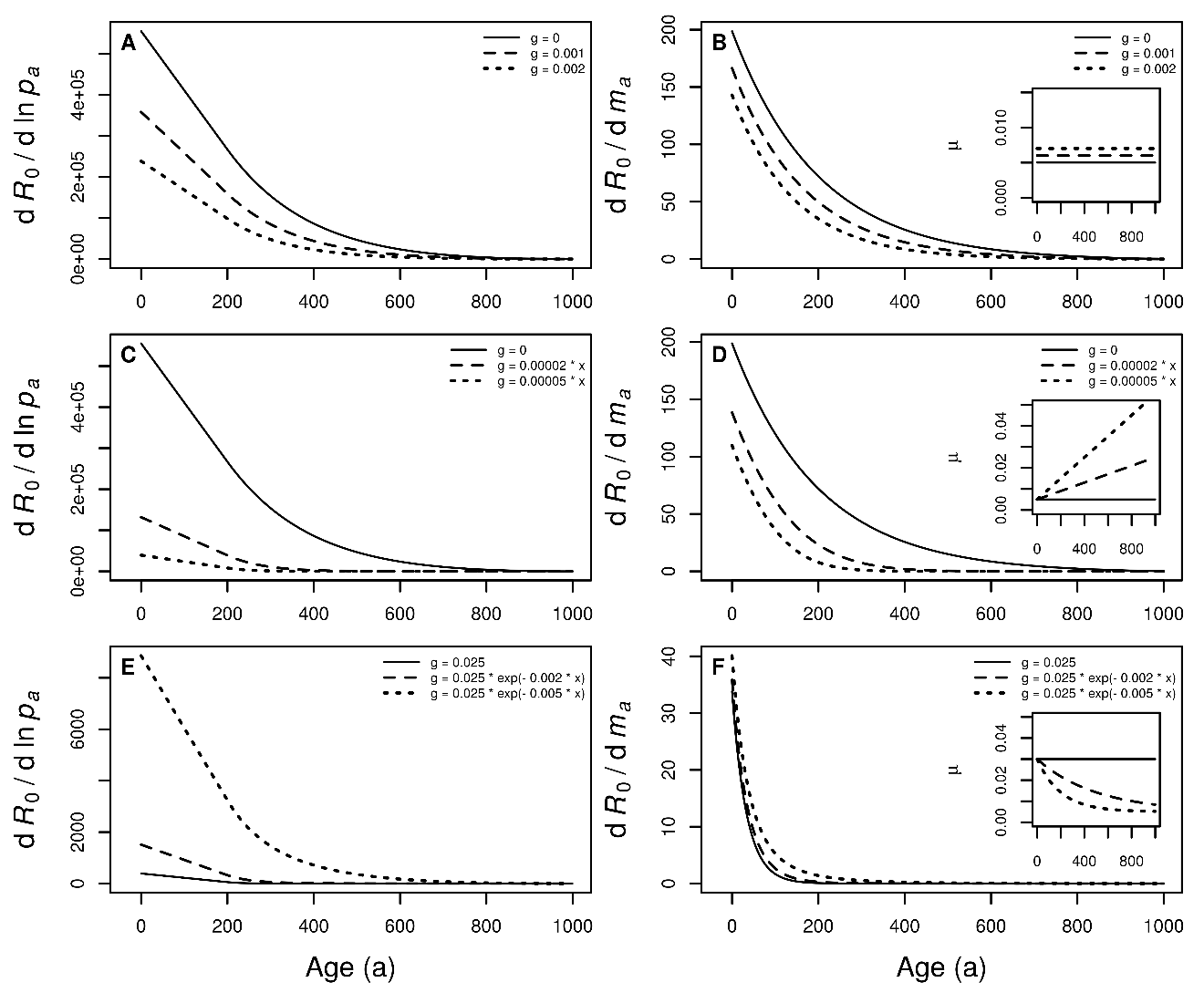


**Figure A2. Selection gradients for *R_0_* under different under different extrinsic mortalities. A mutation affects age *a* onwards.**

**AB** – age-independent extrinsic mortality, **CD** – extrinsic mortality increasing with age, and **EF** – extrinsic mortality decreasing with age. Each panel is characterized by the same *l_x_* and *m_x_* vectors as corresponding panel in **Fig. 1.** Inserts show the total age-specific mortality calculated as $\mu_{x}=-\text{ln}p_{x}+g(x)$, where *g* (extrinsic mortality) is delivered in figures’ legends. For further details see description of **Fig. A1.**

1. **Inserts**


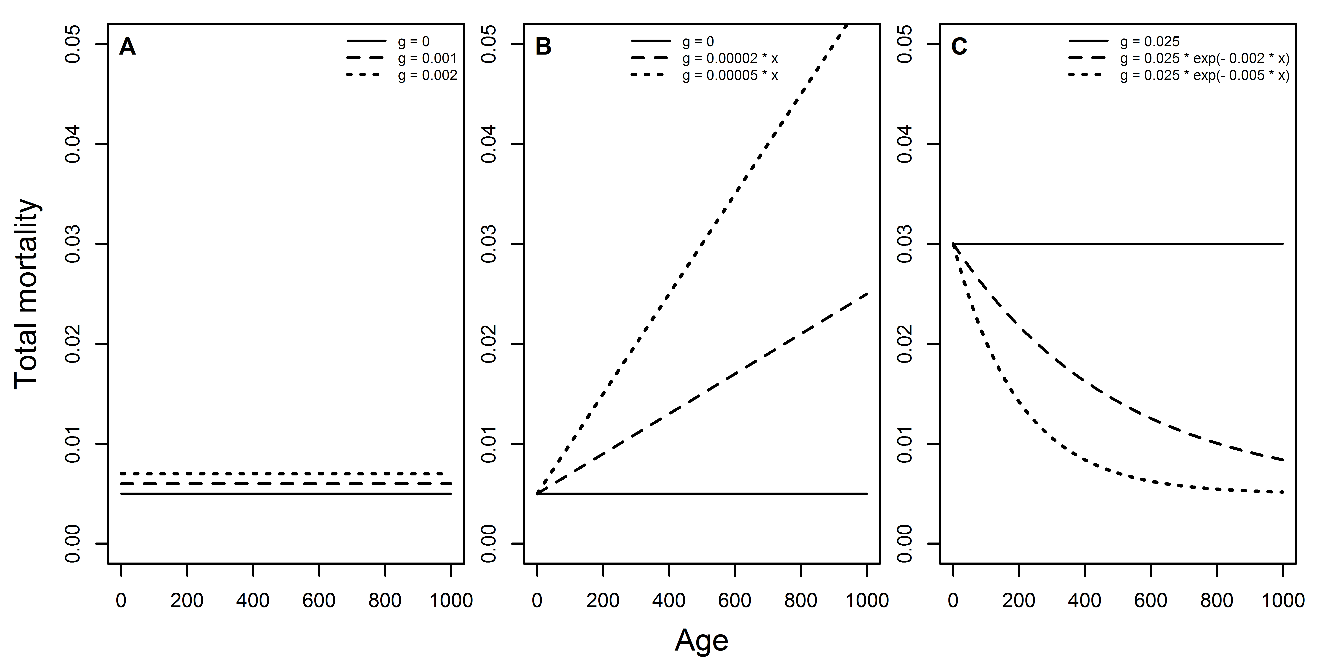


**Figure A3. Total mortality for considered examples (Fig. 1, 2, A1, A2 inserts).** **A** – age-independent extrinsic mortality (**Fig. 1AB** and **Fig. 2AB**), **B** – extrinsic mortality increasing with age (**Fig. 1CD** and **Fig. 2CD**), **C** – extrinsic mortality decreasing with age (**Fig. 1EF** and **Fig. 2EF**). The total age-specific mortality was calculated as $\mu_{x}=-\ln p_{x}+g(x)$, where *g* is delivered in figures’ legends.

1. **Continuous forms of equations used in the manuscript**

**(1C)** $1=\int_{0}^{\infty} e^{-rx}l_{x}m_{x}dx$

**(2C)**$\frac{dr}{d\ln p_{a}}=\frac{1}{T}\int_{a}^{\infty} e^{-rx}l_{x}m_{x}dx$

**(4C)** $T=\int_{0}^{\infty} xe^{-rx}l_{x}m_{x}dx$

**(6C)** $R_{0}=\int_{0}^{\infty} l_{x}m_{x}dx$

**(7C)** $\frac{dR_{0}}{d\ln p_{a}}=\int_{a}^{\infty} l_{x}m_{x}dx$

**(10C)** $V_{x}=\frac{e^{rx}}{l_{x}}\int_{x}^{\infty} e^{-ry}l_{y}m_{y}dy$

**(11C)** $V_{0}=\int_{0}^{\infty} e^{-ry}l_{y}m_{y}dy$
